# Antipsychotic treatment and cognitive function in patients with schizophrenia

**DOI:** 10.1192/j.eurpsy.2023.328

**Published:** 2023-07-19

**Authors:** M. A. Tumova, A. A. Stepanova, M. G. Yanushko, A. P. Kotsyubinsky, M. V. Ivanov

**Affiliations:** 1 Biological therapy of the mentally ill; 2 Department of Biopsychosocial Rehabilitation, V.M. Bekhterev National medical research center psychiatry and neurology, Saint Petersburg, Russian Federation

## Abstract

**Introduction:**

It is known that cognitive impairment is one of the main symptoms of schizophrenia, which determines the functional outcome. The question of the effect of antipsychotics on the cognitive functions of these patients is still unresolved. Cognitive impairment while taking antipsychotics is thought to be mostly related to extrapyramidal abnormalities. In practice, it is difficult to distinguish what causes a patient’s complaints of cognitive decline. Is it related to taking the medication? Or a worsening mental state? Age, lifestyle, etc.?

**Objectives:**

We analyzed the relationship of cognitive impairment with the severity of extrapyramidal symptoms, mental status gravity, age, and dose of antipsychotic and cholinergic medication at weeks 2 and 8 of treatment.

**Methods:**

We examined 37 patients with schizophrenia on stable antipsychotic treatment at weeks 2 and 8 of therapy. Thirty patients received a 2nd-generation antipsychotic, and seven patients received a 1st-generation antipsychotic. The anticholinergic drug was trihexyphenidyl. The antipsychotic dose was estimated in olanzapine equivalent. Extrapyramidal symptoms were assessed by The Scale for Extrapyramidal Symptoms (SAS), severity of mental condition was rated by The Positive and Negative Syndrome Scale (PANSS), cognitive function was measured by The Brief Assessment of Cognition in Schizophrenia (BACS).

**Results:**

As previously described, patients with more severe extrapyramidal symptoms tended to have lower BACS composite scores (rxy = -0.318, p-value = 0.055) at week 8 of therapy. The total score on the SAS scale, as expected, only negatively correlated with scores on the Token Motor Task test (rxy = -0.412, p-value = 0.011) at the 8th week of therapy. There were also negative correlations between Token Motor Task scores and trihexyphenidyl dose (rxy = -0.496, p-value = 0.002). At both weeks 2 and 8, there was a negative relationship between age and Symbol Coding scores (rxy = -0.387, p-value = 0.018; rxy = -0.35, p-value = 0.034, respectively). Verbal Fluency scores were lower in patients with high scores on the PANSS excitement component and at week 2 (rxy = -0.42, p-value = 0.01), this trend continued at week 8 (rxy = -0.31, p-value=0.063) . Tower of London scores were negatively associated at week 8 with cognitive and positive PANSS scores (rxy = -0.46, p-value = 0.004; rxy = -0.336, p-value = 0.042, respectively).

**Conclusions:**

Thus, we have demonstrated that cognitive impairment in patients with schizophrenia is associated with various factors, and not only antipsychotic treatment.

**Disclosure of Interest:**

None Declared

